# Supplementary material for: Socioeconomic differences in inpatient care expenditure in the last year of life among older people: a retrospective population-based study in Stockholm County
Source: BMJ Open. 2022 Jul 8;12(7):e060981. doi: 10.1136/bmjopen-2022-060981 (PMC9272112; doi:10.1136/bmjopen-2022-060981)
Supplement: Author's manuscript [file bmjopen-2022-060981.draft_revisions.pdf]

BMJ Open is committed to open peer review. As part of this commitment we make the peer review history of every article we publish publicly available.

When an article is published we post the peer reviewers' comments and the authors' responses online. We also post the versions of the paper that were used during peer review. These are the versions that the peer review comments apply to.

The versions of the paper that follow are the versions that were submitted during the peer review process. They are not the versions of record or the final published versions. They should not be cited or distributed as the published version of this manuscript.

BMJ Open is an open access journal and the full, final, typeset and author-corrected version of record of the manuscript is available on our site with no access controls, subscription charges or pay-per-view fees (http://bmjopen.bmj.com).

If you have any questions on BMJ Open's open peer review process please email info.bmjopen@bmj.com

BMJ Open

Socio-economic differences in Inpatient Care Expenditure in the Last Year of Life among older people in Stockholm County

Journal:	*BMJ Open*
Manuscript ID	bmjopen-2022-060981
Article Type:	Original research
Date Submitted by the Author:	14-Jan-2022
Complete List of Authors:	Doheny, Megan; Karolinska Institutet, Global Public Health<br>Schön, Pär; Aging Research Center<br>Orsini, Nicola; Karolinska Institutet, Global Public Health<br>Walander, Anders; Region Stockholm, Center for Epidemiology and Community Medicine<br>Burström, Bo; Karolinska Institutet, Global Public Health<br>Agerholm, J; Aging Research Center
Keywords:	SOCIAL MEDICINE, HEALTH SERVICES ADMINISTRATION & MANAGEMENT, Health policy < HEALTH SERVICES ADMINISTRATION & MANAGEMENT, PUBLIC HEALTH

SCHOLARONE™
Manuscripts

I, the Submitting Author has the right to grant and does grant on behalf of all authors of the Work (as defined in the below author licence), an exclusive licence and/or a non-exclusive licence for contributions from authors who are: i) UK Crown employees; ii) where BMJ has agreed a CC-BY licence shall apply, and/or iii) in accordance with the terms applicable for US Federal Government officers or employees acting as part of their official duties; on a worldwide, perpetual, irrevocable, royalty-free basis to BMJ Publishing Group Ltd ("BMJ") its licensees and where the relevant Journal is co-owned by BMJ to the co-owners of the Journal, to publish the Work in this journal and any other BMJ products and to exploit all rights, as set out in our licence.

The Submitting Author accepts and understands that any supply made under these terms is made by BMJ to the Submitting Author unless you are acting as an employee on behalf of your employer or a postgraduate student of an affiliated institution which is paying any applicable article publishing charge ("APC") for Open Access articles. Where the Submitting Author wishes to make the Work available on an Open Access basis (and intends to pay the relevant APC), the terms of reuse of such Open Access shall be governed by a Creative Commons licence – details of these licences and which Creative Commons licence will apply to this Work are set out in our licence referred to above.

Other than as permitted in any relevant BMJ Author's Self Archiving Policies, I confirm this Work has not been accepted for publication elsewhere, is not being considered for publication elsewhere and does not duplicate material already published. I confirm all authors consent to publication of this Work and authorise the granting of this licence.

For peer review only

Socio-economic differences in Inpatient Care Expenditure in the Last Year of Life among older people in Stockholm County

Megan Doheny

Department of Global Public Health, Karolinska Institutet, Stockholm, Sweden

Pär Schön

Aging Research Center, Karolinska Institutet - Stockholm University, Stockholm, Sweden

par.schon@ki.se

Nicola Orsini

Department of Global Public Health, Karolinska Institutet, Stockholm, Sweden;

nicola.orsini@ki.se

Anders Walander

Center for epidemiology and community medicine, Region Stockholm, Sweden.

anders.walander@regionstockholm.se

Bo Burström

Department of Global Public Health, Karolinska Institutet, Stockholm, Sweden.

Center for epidemiology and community medicine, Region Stockholm, Sweden.

bo.burstrom@ki.se

Janne Agerholm

Aging Research Center, Karolinska Institutet - Stockholm University, Stockholm, Sweden

janne.agerholm@ki.se

Corresponding author: Megan Doheny
Orcid !D: 0000-0002-5640-1239
Address: Department of Global Public Health, Karolinska Institutet, Widerströmska plan 3, 171 71, Stockholm, Sweden.
E-mail: megan.doheny@ki.se
Telephone: +46 7 2548 6112

For peer review only

For peer review only

For peer review only - http://bmjopen.bmj.com/site/about/guidelines.xhtml

ABSTRACT

Objectives: This study aimed to investigate the association between inpatient care expenditure (ICE) and socio-economic position, and the effect of demographic factors, health status, healthcare, and social care utilization on ICE in the last year of life.

Design: Resorptive population-based study

Setting: Stockholm County.

Participants: Decedents ≥65 years in 2015 (N=13,538).

Outcome: ICE was calculated individually for the month of, and 12 months preceding death using healthcare register data from 2014 and 2015. ICE included the costs of admission and treatment in inpatient care for acute somatic, geriatric, surgical and psychiatric departments adjusted for the price level in 2018.

Results: Those in higher income groups incurred higher ICE (10,032SEK CI95% 4,714; 15,350) at the 75th percentile compared to the lowest income group and there was a social gradient in ICE incurred (45,307SEK CI95% 12,055; 79,559) at 95th percentile. Higher ICE was driven by greater morbidity and use of Emergency Department care, while lower ICE was associated with older age, country of birth and months in institutional care.

Conclusion: Gaining insight into patterns of healthcare spending in the last year of life has important implications for policy, particularly as socio-economic differences were visible in ICE at a time of greater care need for all. Future policies should focus on engaging in advanced care planning and strengthening the coordination of care for older people.

Strengths and Limitations

- The quantile regression (QR) has has flexible assumptions compatible with cost data and assesses the relationship between SEP and other factors across the distribution of ICE.
- It was only feasible to measure ICE in this study; however, this does not provide a complete overview of total costs of care accrued.
- There are difficulties in measuring need of care and changes in need during the last year of life.

Keywords

End-of-life, inpatient care expenditure, health and social care need, socio-economic differences, ageing in place.

Abstract: 294 words

Main text: 3,458 word

For peer review only

INTRODUCTION

An ageing population is often considered the main driver of increasing healthcare expenditure due to increasing prevalence of multi-morbidity (2+ chronic conditions) and more complex health problems [1]. However, age itself is not the main driver of healthcare expenditure but rather proximity to death, as a large proportion of all deaths occur among those 65 years and older [2-4], and a substantial proportion of an individual's lifetime healthcare expenditure occurs during the last year of life [5].

Patterns of healthcare utilisation in the last year of life often involve high rates of acute hospital-based services [6]. A country comparison study of the composition of end-of-life (EOL) expenditure across nine different countries showed that expenditure on hospital-based inpatient care accounted for the largest proportion of total expenditure, followed by the utilisation of social care [7]. Additionally, a Swedish study also observed that inpatient care specialties accounted for 80% of state expenditure on healthcare in the last year of life [8].

Often high spending on healthcare among older persons is not due to the use of more expensive life-saving treatments, but rather due to spending on treating persons with multiple chronic conditions [5]. Generally, healthcare utilisation and as such healthcare expenditure is largely influenced by the need of care, and health status which varies by socio-economic position (SEP). Frequently, older persons with lower SEP experience poorer health and have greater need of hospital-based care at the EOL which would lead to decedents with lower SEP incurring higher ICE [9]. However, previous studies have observed socio-economic differences in healthcare expenditure, where persons with higher SEP have higher expenditure compared to those with lower SEP [8, 10-13].

In Sweden, the health and social care system is universal and mainly financed through taxation, where the patient pays a small fraction of the total cost of care. Further, the provision of care is based on the principle of "equal access for equal need" regardless of an individual's age, sex, or economic resources [14]. According to a recent OECD report Sweden has one of the highest average healthcare expenditures per capita, 4,676 euros. On the other hand, there is on average 2.1 hospital beds per 1,000 persons in Sweden which is among the lowest in OECD [15]. In Sweden, the 21 Regions are responsible for providing health and medical care while the 290 municipalities are responsible for providing social care for older people (home-help services and institutional care). In institutional care there is onsite health and medical care being provided by nurses, assistant nurses, and Primary Health Care (PHC) doctors, in

For peer review only - http://bmjopen.bmj.com/site/about/guidelines.xhtml

addition to care personnel that provide daily personal care (bathing, eating, and hygiene), social activities and companionship in institutional care settings [16]. Home-help services can be offered around the clock and provides two main types of support: household tasks (e.g., cleaning and laundry, shopping, cooking or meals on wheels) and personal care. In parallel to the reduction in hospital-beds, there has been a similar decrease in the number of places in municipal institutional care, in Sweden. These changes have been brought about as cost containment strategies driven by the "Ageing in place" policy, which has resulted in an increasing number of older persons dependent on receiving care and support in their own homes [16].

Increasing the knowledge of the drivers of ICE, and investigating whether there are socio-economic differences is important considering the ageing population with greater care needs and the goal of providing equitable care. This study aimed to investigate the association between inpatient care expenditure (ICE) and socio-economic position and the effect of other socio-demographic factors, health status, healthcare, and social care utilization on ICE in the last year of life.

METHODS

The study population was retrospectively identified, including those 65 years and older that died in the calendar year 2015 in Stockholm County (N=13,538). The last year of life was defined as the month of death plus the 12 months preceding the month of death. The Cause of Death register used to identify the study population using the year and month, to measure the age of death and the underlying cause of death [17]. The underlying cause of death categories were cancer-related, cardiovascular-related, neurodegenerative-related (including dementia, Alzheimer's, Parkinson's disease) and other causes [18].

Data sources

The Region Stockholm Healthcare Administrative Register (VAL by Swedish acronym) was used to measure the outcome Inpatient Care Expenditure (ICE) which included the costs of admission (planned and unplanned) and treatment in inpatient care for acute somatic, geriatric, surgical and psychiatric departments. ICE was calculated for 2014 and 2015, adjusted for the price level in 2018 in Swedish Kronor (SEK), (10 SEK = 0.98 euros). The outcome was total ICE for each decedent measured as their total inpatient expenditures accrued from their month of death plus 12 months preceding death.

For peer review only - http://bmjopen.bmj.com/site/about/guidelines.xhtml

Socio-demographic characteristics of decedents were obtained from the Longitudinal Integration database for Health Insurance and Labour Market Studies (LISA). This register contains a collection of variables from different population registers linked individually via encrypted serial numbers. We measured sex, country of birth, living situation, and income in LISA. Sex was grouped as male and female. Country of birth was dichotomized as born in Sweden and born outside of Sweden. Living situation was measured for decedents living in the community and categorised as cohabiting or living alone. SEP was measured using income and assessed using the net annual equalized individual household income from 2012 and then ranked into income quintiles. Those missing a measure for income were excluded from the main analysis (n=56).

Healthcare utilisation during the last year life was obtained from VAL. PHC use was measured by the number of visits to GPs in outpatient care, categorised into 0-5 visits, 6-10 visits and >10 visits. Emergency department (ED) visits were defined as a registered emergency care visit to acute care hospitals in Stockholm County, and categorized into 0-1 visits, 2-3 visits and 4+ visits (frequent ED use). Both measures included in the regression analyses as continuous count variables.

Home-healthcare is a service provided in the patient's home, there are two levels, basic and advanced, which are provided based on medical need. Basic home-healthcare is provided to those that require simpler medical interventions up to nursing-level, such as assistance with taking medication, dressing of wounds, catheter replacement, or to those who have difficulties visiting GPs. Advanced home-healthcare is provided to seriously ill patients in need and involves medical procedures performed at home rather than in a hospital setting, often provided to cancer patients or those with complex EOL care needs [19].

To measure morbidity prior to death we calculated the Charlson Comorbidity Index (CCI) using registered diagnoses in VAL prior to the last year of life. The CCI assigns scores ranging from 1 to 6 to morbidities based on the severity of illness and risk of death [20], the CCI was calculated per decedent and was described in categories: 1-2 score, 3-4 score, and 5+ score, and was included as a continuous variable in regression analysis.

The Swedish Social Services Register was used to define municipal social care utilization. We identified those receiving home-help (personal care and/or domestic services) in their own homes, individuals registered as living in an institution for the entire last year of life, and individuals that were receiving home-help and transitioned into institutional care in the last

For peer review only - http://bmjopen.bmj.com/site/about/guidelines.xhtml

year of life. In the regression analysis the utilisation of home-help and institutional care was measured by number of months of use in the last year of life.

Patient and public involvement

The data used in this study was based on encrypted personal numbers so that the individuals in the study population are not identifiable.

Statistical Analysis

A quantile regression (QR) was selected to investigate how ICE varied between income groups (SEP) and to identify factors that affect the ICE incurred in the last year of life, because the dependent variable ICE has a positively skewed distribution with extreme outliers [21, 22]. The QR model estimates the change in a specified quantile (i.e., percentiles) of the conditional distribution of ICE due to a unit change in the independent variable. The QR was used to assess which independent variables are associated with quantiles of expenditure and has been recommended compared to alternative approaches in previous studies [23-25]. We estimated ICE at the 50th, 75th, and 95th percentiles, adjusting for the following explanatory variables (income, sex, age, country of birth, CCI score, ED use, home-healthcare and municipal social care use). These percentiles were selected as it was presumed that the rate of change in ICE per unit change in an explanatory variable $(\Delta ICE/\Delta x)$ would be progressively greater in the higher percentiles. There was n=2,912 decedents who incurred zero ICE in the last year of life. A logistic regression model was used assess whether there were socio-economic differences among those that incurred zero ICE in the last year of life.

RESTULTS

There were N=13,538 decedents included in the study population described in **table 1**. Most decedents (51.7%) were 85+ years, (54.2%) female and (25.7%) in income group 3. Most decedents in the community were living alone (63.4%), and 18.5% were born outside of Sweden. There were 38.8% of decedents who had a cardiovascular-related death and 25.3% with a cancer-related underlying cause of death. During the last year of life, decedents had on average 4.5 PHC visits and 2.7 ED visits. Further, 28.6% of decedents frequently attended ED care (4+ visits) in the last year of life. Most decedents utilized municipal social care, 32.5% received home-help services, 28.3% were in institutional care, and 10.5% had transition from receiving home-help into an institution during the last year of life.

For peer review only - http://bmjopen.bmj.com/site/about/guidelines.xhtml

Table 1: Description of decedents 65+ years, and the distribution of of Inpatient Care Expenditure (ICE) in the last year of life

	N	(%)	Percentiles of Inpatient Care Expenditure (SEKs) (median) 50th percentile	75th percentile	(highest) 95th percentile
	13,538	100	129,886	279,152	644,596
**Income group**					
Group 1 (Lowest)	2,647	19.6	139,401	281,968	633,349
Group 2	3,160	23.3	131,404	284,388	656,418
Group 3	3,475	25.7	120,833	260,599	617,288
Group 4	2,517	18.6	132,311	291,504	660,780
Group 5 (highest)	1,683	12.4	132,203	287,722	669,124
**Age in years**	83.8(9.0)				
65 - 74 years	2,672	19.7	161,587	358,466	903,567
75 - 84 years	3,875	28.6	158,874	321,478	721,371
85+ years	6,991	51.7	109,658	231,026	506,885
**Sex**					
Male	6,204	45.8	147,442	306,065	702,300
Female	7,334	54.2	116,217	257,460	591,401
**Country of birth**					
Sweden	10,963	81.5	131,695	281,814	647,807
Other	2,489	18.5	128,031	275,844	624,877
**Living situation**					
Cohabiting	3,544	36.6	168,215	322,015	700,553
Alone	6,132	63.4	115,251	259,849	613,316
**Healthcare utilisation**					
**Average no. PHC visits**	4.5 (6.9)				
0–5 PHC visits	9,753	72.0	104,608	239,889	585,186
6–10 PHC visits	2,011	14.9	191,834	345,298	735,614
>10 PHC visits	1,774	13.1	216,465	387,845	804,472
**Average no. ED visits**	2.7 (3.0)				
0–1 ED visits	5,460	40.3	25,433	88,392	299,568
2–3 ED visits	4,208	31.1	156,467	248,957	522,653
>4 ED visits	3,870	28.6	317,664	481,791	870,365
**Home-health care**					
Basic care	3,602	26.6	225,700	382,229	759,464
Advanced care	1,651	12.2	202,885	355,234	718,165
**Social care utilisation**					
Independent	3,863	28.5	125,057	269,586	696,304
Home-help	4,393	32.5	203,872	359,895	721,371
Institutional care	3,862	28.5	41,823	130,698	392,649
Transition	1,420	10.5	202,336	346,695	710,872
**Months with home-help**	8,2(4.9)				
**Months in institutional care**	9.0(3.8)				
**Health status and Underlying cause of death**					
**CCI score**	3.1(2.6)				
0 - 2 score	6,878	50.8	60,216	163,026	451,067
3 - 4 score	2,873	21.2	188,170	341,629	697,367
5 + score	3,787	28.0	236,137	406,819	805,941
**Cause of Death**					
Cancer-related	3,425	25.3	195,908	343,042	681,738
Cardiovasuclar-related	5,221	38.6	119,816	274,621	654,265
Neurodegenerative related	1,995	14.7	45,393	143,758	396,939
Other	2,897	21.4	132,820	288,010	686,942

*Variables living situation and home-healthcare utilization are measures applicable to those living in the community for in the last 12-months of life (n=9,67

For peer review only - http://bmjopen.bmj.com/site/about/guidelines.xhtml

Those in the lowest income group incurred higher costs at the 50th percentile (139,401 SEK) compared to other income groups, while at the 75th and 95th percentile those in the highest income group incurred higher ICE (287,722 SEK at 75th) and (669,124 SEK at 95th) compared to the lowest income group (281,968 SEK at 75th) and (633,349 SEK at 95th) respectively. There was an inverse relationship between age and ICE, where those 85+ years incurred lower ICE compared to those 65-74 years. Females incurred lower ICE compared to males at the 50th, 75th and 95th percentiles, a similar pattern could be observed among those living alone and those born outside of Sweden.

Decedents that had more visits to PHC and other outpatient care services incurred higher ICE. Those receiving basic home-healthcare incurred higher ICE compared to those receiving advanced home-healthcare. Decedents receiving home-help services or that transitioned to institutional care incurred higher ICE compared to those independent. Persons with cancer-related deaths incurred higher ICE at the 50th, 75th and 95th percentiles, while those with neurodegenerative-related cause of death incurred lower ICE.

Quantile Regression

Table 2 contains the estimates from the QR analysis. We observed socio-economic differences in ICE incurred at the 75th and 95th percentile by income group. Those in the second, third- and fourth -income group incurred higher ICE at the 75th percentile compared to the lowest income group. There was a social gradient in the ICE incurred at the 95th percentile as those in higher income groups incurred higher ICE. Those in the highest income group incurred (45,307SEK CI95% 12,055; 79,559) higher ICE compared to those in the lowest income group at the 95th percentile.

.

For peer review only

Table 2: Quantile regression (QR) estimates conditional on the 50th (median), 75th and 95th (high-cost patients). of the distribution of Inpatient Care Expenditure in the last year of life among all decedents 65+ years in Stockholm County.

	**50th percentile**		**75th percentile**		**95th percentile**	
	**Coefficient (SEK)**	**CI 95%**	**Coefficient (SEK)**	**CI 95%**	**Coefficient (SEK)**	**CI 95%**
Income group						
Group 1 (lowest)	ref		ref		ref	
Group 2	1,925	-474 – 4,323	10,032	4,714 – 15,350	41,720	14,824–68,615
Group 3	-520	-3,201 – 2,161	9,354	3,273 – 15,436	43,252	19,263–67,240
Group 4	304	-2,468 – 3,076	11,704	4,495 – 18,913	42,762	11,875–73,650
Group 5 (highest)	-912	-3,805 – 1,981	8,360	-817 – 17,537	45,307	12,055–78,559
Age in years	-304	-469 – 139	-1,672	-2,154 – -1,190	-9,761	-12,070– -7,452
Sex						
Male	ref		ref		ref	
Female	2,635	-145 –5,414	5,126	-1,384 – 11,636	11,614	-11,220–34,449
Country of birth						
Sweden	ref		ref		Ref	
Outside of Sweden	-2,736	-4,367 – -1,104	-11,318	-15,785 – -6,851	-21,054	-41,726–-382
CCI score	12,358	11,180 – 13,537	16,720	14,999 – 18,441	20,333	12,673–27,993
ED visits	42,586	41,002 – 44,170	57,362	54,463 – 60,260	71,995	64,442–79,549
Home-healthacre						
None	Ref		ref		ref	
Basic care	31,017	23,791 – 38,243	36,040	21,050 – 51,029	15,299	-23,046 – 55,643
Advanced care	11,412	2,777 – 20,048	34,179	20,382 – 47,976	-32,009	-82,581– 18,563
Months of home-help	475	-54 – 1,005	456	-677 –1,589	-4,216	-7,386 – -1,045
Months of institutional care	-945	-1,117 – -774	-2,074	-2,741– -1,408	-11,593	-15,136 – -8,050
Intercept	24,620	11,015 – 38,224	163,558	122,071 – 205,545	1,061,896	855,819 –1,267,972

*QR model adjusted for income group (ref=lowest income group 1), age in years, sex (ref=male), country of birth (ref=Sweden), CCI score, ED visits, Home-healthcare (ref=no care in the community), months in institutional care and months receiving home-help.

For peer review only

For peer review only - http://bmjopen.bmj.com/site/about/guidelines.xhtml

Increasing age in years was associated with lower ICE at the 50th, 75th and 95th percentile. There were no significant differences between sexes. Decedents born outside of Sweden incurred lower ICE at the 50th (-2,736SEK CI95%-4,367; -1,104) and at the 75th percentile (-11,318SEK CI95% -15,785; -6,851) compared to decedents born in Sweden. Greater morbidity and more visits to ED care were positively associated with incurring higher ICE. ED visits were a significant driver for higher ICE, an increase in the number ED visits was associated with (71,995 SEK CI 95% 64,442; 79,549) higher ICE at the 95th percentile. Those receiving basic home-healthcare incurred higher ICE at the 50th and 75th percentiles, there was similar pattern with advanced home healthcare but slightly lower at the 75th percentile (34,179 SEK CI95% 21,050; 51,029). Months in institutional care were associated with incurring lower ICE across the distribution, while additional months with home-help were associated with lower ICE (-4,216 SEK CI95% -7,1386; -1,045) at the 95th percentile

Zero Expenditure

There were 2,912 decedents that incurred zero ICE, 56.6% of decedents with zero costs resided in institutional care and 29.9% lived independently. Those with zero cost living in the community had an average age of death of 78.4 years, were mostly male, 26.5% born outside of Sweden, utilised more PHC than ED care in the last year of life and 53.2% had cardiovascular-related death. Table 3 shows models estimating odds of incurring zero ICE among decedents that were living in the community, there were no socio-economic differences observed in the odds of zero ICE, while those born outside of Sweden and had a cardiovascular-related underlying cause of death had higher odds of zero ICE. Older age and the utilisation of healthcare and social care were associated lower odds of having zero ICE.

Table 3: Logistic regression estimates the odds of incurring zero inpatient care costs in the last year of life.

	**All Zeroes**	**Community living decedents**						
	**N=2,912 (%)**	**N=1,254 (%)**	Model 1			Model 2		
			**OR**	**CI95%**		**OR**	**CI95%**	
Income group								
(lowest) Group 1	17.20%	20.9	ref			ref		
Group 2	24.10%	21.5	1.02	0.77	1.36	0.96	0.74	1.25
Group 3	27.90%	24.6	0.93	0.70	1.24	0.90	0.69	1.18
Group 4	18.50%	19.4	0.95	0.70	1.28	0.95	0.72	1.26
(highest) Group 5	12.20%	13.6	1.01	0.72	1.40	0.99	0.73	1.35
**Age in years**	84.9 (9.9)	78.6 (9.1)						
65-74 years	19.40%	39.60%	ref			ref		
75-84 years	22.70%	30.30%	0.86	0.68	1.07	0.87	0.71	1.08

For peer review only

85 + years	57.80%	30.10%	0.71	0.56	0.90	0.80	0.64	1.00
**Sex**								
Male	39.20%	54.30%	ref			ref		
female	60.80%	45.70%	0.90	0.75	1.09	0.96	0.80	1.15
**Living situation**								
Cohabiting			ref			ref		
Living alone	69.10%	69.10%	1.13	0.91	1.41	1.23	1.00	1.50
**Country of Birth**								
Sweden			ref			ref		
Outside of Sweden	19.50%	26.50%	1.42	1.13	1.77	1.39	1.13	1.70
**CCI score**	1.1(1.6)	1.0 (1.9)	0.59	0.56	0.62			
**Healthcare utilisation**								
Average no. ED visits	0.3 (0.7)	0.4 (0.8)	0.17	0.15	0.19	0.14	0.13	0.16
Average no. PHC visits	1.9 (4.8)	4.3 (6.5)	0.99	0.97	1.00			
**Home-healthcare**								
none			ref			ref		
Basic	5.20%	12.00%	0.55	0.42	0.71	0.44	0.35	0.57
Advanced	4.30%	10.00%	1.04	0.77	1.42	0.70	0.51	0.95
**Cause of death**								
Other	21.60%	25.20%				ref		
Cancer	8.40%	15.30%				0.29	0.22	0.39
Cardiovascular	41.30%	53.20%				1.30	1.05	1.60
Neurodegenerative	28.70%	6.30%				1.21	0.84	1.72
**Social care utilisation**								
Independent	29.90%	68.80%						
Home-help	10.50%	24.20%						
Institutional care	56.60%	.						
Transition	3.00%	7.00%						
**Months with home-help**			0.98	0.96	1.00	0.97	0.95	0.99

*column one the description of those with zero ICE in the last year of life in proportions (%) and mean and standard deviation.*OR=odds ratio, CI95% =95% confidence interval ref=indicating the reference group, *Logistic regression models stratified into the group of persons with zero costs living in the community n=1,254 for the most of their last year of life.

*Model 1 is adjusted for (income group, age groups, sex, country of birth, living situation, phc visits, ED visits, home-healthcare, CCI score, months with home-help).

*Model 2 is adjusted for (income group, age groups, sex, country of birth, living situation, phc visits, ED visits, home-healthcare, underlying cause of death, months with home-help).

For peer review only

For peer review only - http://bmjopen.bmj.com/site/about/guidelines.xhtml

DISCUSSION

This study investigated the socio-economic differences in inpatient care expenditure (ICE) in the last year of life and, assessed the effect of other demographic factors, health status, healthcare and social care utilisation had on ICE. We observed that decedents in the higher income group incurred higher ICE at the 75th percentile, and there was a social gradient in ICE at the 95th percentile with higher ICE in the higher income groups. Older age was associated with incurring lower ICE across the distribution. Greater morbidity and visits to ED care were positively associated with incurring higher ICE across the distribution. Months in institutional care was associated with incurring lower ICE overall, while months with home-help were associated with lower ICE at the 95th percentile.

Based on findings from earlier studies, we expected that older people with lower SEP due to greater need would utilise more healthcare and incur greater ICE [8-13, 21]. Previous studies from the UK observed that persons in the most deprived quintile incurred higher inpatient care costs than the least deprived [12], similar findings were observed in other studies on those 65 years and older [11, 13]. Our findings are akin with a previous Swedish study that found the persons in the highest income group had higher healthcare expenditure in the last year of life compared to the lowest [8]. In contrast to this study, Hanratty et al included adults of all ages and measured total healthcare expenditure (inpatient and outpatient costs) and yet, we observed similar socio-economic differences despite these deviations in study population and outcome [8].

Poorer health and functioning in the last year of life should be the main determinant of hospital use and subsequent expenditure on care. A systematic review and meta-analysis found that socio-economic differences in EOL healthcare expenditure can vary based on adjustment for need of care [26]. We took this into consideration and included the CCI score as an indicator of "need" measured prior to the last year of life. The CCI score has been demonstrated to be effective at predicting persons who will incur high healthcare costs [27]. The socio-economic differences were observed at the top 5% of the distribution and remained after adjusting for need, but whether these differences are indicating inequities in access or treatment cannot be determined in this study.

The socio-economic differences observed in this study may have many explanations. It could be due to more affluent and better educated individuals or their families being better equipped to navigate the healthcare system and advocate for more extensive or expensive EOL care.

For peer review only - http://bmjopen.bmj.com/site/about/guidelines.xhtml

However, this pro-rich bias of hospital care has had mixed findings, a Scottish study that found there were no differences in the costs incurred by SEP once hospitalized, though differences were observed in when persons from more deprived areas reached hospital care [28]. Further, a Swedish study found that decedents 65+ years with tertiary education were more likely to die in hospital compared to those primary education [29]. This raises the question of appropriateness of EOL care if socio-economic differences in ICE is due to older people with high SEP being more likely to die in hospital, as a systematic review reported that people prefer to die in their own homes, even as illness progresses [30].

Age was associated with incurring lower ICE in the last year of life, this finding is consistent with previous studies [3,4,6-13] and in line with the "Red Herring" theory, which stipulates that as age increases healthcare expenditure decreases and social care expenditure increases [2, 3]. Decedents residing in institutional care incurred lower ICE and a large proportion incurred zero ICE. This result might be due to the around-the-clock presence of health professionals and caregivers in institutional care settings, which may facilitate better EOL care. Previous studies observed that decedents living independently were more frequently hospitalised and had higher healthcare costs compared to those in institutional care after standardizing for similar care needs [31]. Similarly, a Swedish study found that older people living in their own homes were hospitalized more frequently in the last 10 weeks of life [32].

Older people are now are increasingly "ageing in place", and their care needs shall be met at home. There is a greater need for advanced care planning near the EOL, as the majority of decedents in our study were receiving social care services and even those receiving home-healthcare incurred higher ICE. A Swedish report comparing healthcare systems in ten different countries, highlighted that Sweden had deficits in care planning at the end-of-life [33]. ED care visits were a driver of higher ICE, possibly due to subsequent unplanned hospitalisations and treatment in inpatient care [32]. However, if care received in the home is sufficiently meeting needs, as found in a systematic review, receiving appropriate palliative home-based care should lower ED use among dying patients [34]. Nevertheless, a previous Swedish study found that receiving home-healthcare was associated with frequent ED use [35].

Additionally, care transitions that occur in the last year of life are stressful for patients and family members as well as being costly and difficult to organise in the care system. A Canadian study found that high-cost acute care users had multiple care transitions during the last year of life, and had longer hospital stays because of lack of places in institutional care or

For peer review only - http://bmjopen.bmj.com/site/about/guidelines.xhtml

due to lack of availability of homecare to discharge patients [36]. The provision of social care is essential in the last year life and can reduce inpatient care costs. The de-institutionalisation trend might leave some patients in precarious living situations during a difficult time, as the overall length of stay in institutions is decreasing with a large proportion of people moving into institution and dying shortly afterward, in Sweden [37].

Strengths and Limitations

A strength of this study is the use of high-quality register data, that allowed us to retrospectively follow our study population and measure their ICE and their utilisation of other health and social care services in last year of life. The QR model allowed us assess the relationship between SEP and other factors across the distribution of ICE from lowest to the highest, as the QR model has flexible assumptions [21, 22]. The Swedish Cause of Death Register provides complete coverage of the population, and we have measures for all decedents [18]. However, as older persons often experience multi-morbidity deciphering the exact cause of death can be difficult especially among the very old [38]. Therefore, we also included the CCI score to indicate need [20], though we are limited in our ability to fully measure an individual's need and the extent that these needs change during the last year of life [5, 27]. Another limitation of this study is that we only focused on expenditure on inpatient care, which does not provide a complete overview of total costs accrued in the EOL, as social care accounts for a substantial proportion of EOL care and there are other costs such as for outpatient visits and pharmaceutical drugs [4, 7]. Future research should use a longitudinal approach and explore the socio-economic differences in patterns of health and social care services in the last year of life and the years prior to understand how socio-economic differences can emerge.

CONCLUSIONS

Most deaths occur in older age, and now most older people with complex health problems are receiving care in their own homes. Gaining insight into patterns of healthcare spending in the last year of life has important implications for policy, as socio-economic differences were visible in ICE at a time of greater care need for all, indicating inequity in end-of-life care. Future policies should focus on engaging in advanced care planning and strengthening the coordination of care in older people homes.

For peer review only - http://bmjopen.bmj.com/site/about/guidelines.xhtml

For peer review only

CONTRIBUTION STATEMENT

MD, PS, AW, BB and JA conceived the idea for, participated in the design and the coordination of this study. MD performed the statistical analysis and drafted the manuscript. MD, JA, NO, PS and BB were all involved in the interpretation of results and the revisions of manuscript drafts. All authors read and approved the submitted manuscript.

FUNDING

The authors disclosed receipt of the following financial support for the research, authorship and/or publication of this article: The research leading to these results was carried out as part of the Social Inequalities in Ageing (SIA) project, funded by NordForsk project no. 74637, and funding was also provided by the Swedish Research Council for Health, Working Life and Welfare (Forte), grant numbers 2017-02155 and 2017-04431.

CONFLICT OF INTEREST

None

ETHICAL APPROVAL

Ethical permissions were obtained from the Regional Ethics Review Board of Stockholm (Dnr 2016/299–31).

PARTICIPANTS CONSENT

Individual consent to participate in this study is waived as this was a population-based study using administrative data.

DATA STATEMENT

The data used will not be shared, as the materials are based on individual data which cannot be shared in accordance with Swedish law and GDPR.

REFERENCES

1. SOU 2016:2. Effektiv vård. Slutbetänkande av En nationell samordnare för effektivare resursutnyttjande inom hälso- och sjukvården. Stockholm 2016 [Effective care. Final report by A national coordinator for more efficient use of resources in health care]
2. Zweifel, P., S. Felder, and M. Meiers (1999), 'Aging of population and health care expenditure: a red herring?', Health Economics, 8(6): 485–496.

For peer review only

3. Polder JJ, Barendregt JJ, van Oers H. Health care costs in the last year of life--the Dutch experience. Soc Sci Med. 2006;63(7):1720-31.
4. Jayatunga W, Lewer D, Shand J, Sheringham J, Morris S, George J. Health and social care costs at the end of life: a matched analysis of linked patient records in East London. Age Ageing. 2019;49(1):82-7.
5. Davis MA, Nallamothu BK, Banerjee M, Bynum JP. Identification Of Four Unique Spending Patterns Among Older Adults In The Last Year Of Life Challenges Standard Assumptions. Health Aff (Millwood). 2016;35(7):1316-23.
6. Ní Chróinín D, Goldsbury DE, Beveridge A, Davidson PM, Girgis A, Ingham N, et al. Health-services utilisation amongst older persons during the last year of life: a population-based study. BMC Geriatr. 2018;18(1):317.
7. French EB, McCauley J, Aragon M, Bakx P, Chalkley M, Chen SH, et al. End-Of-Life Medical Spending In Last Twelve Months Of Life Is Lower Than Previously Reported. HealthAff(Millwood). 2017;36(7):1211-7.
8. Hanratty B, Burström B, Walander A, Whitehead M. Inequality in the face of death? Public expenditure on health care for different socioeconomic groups in the last year of life. J HealthServRes Policy. 2007;12(2):90-4.
9. Davies JM, Maddocks M, Chua KC, Demakakos P, Sleeman KE, Murtagh FEM. Socioeconomic position and use of hospital-based care towards the end of life: a mediation analysis using the English Longitudinal Study of Ageing. Lancet Public Health. 2021;6(3):e155-e63.
10. Cunningham CM, Hanley GE, Morgan SG. Income inequities in end-of-life health care spending in British Columbia, Canada: A cross-sectional analysis, 2004-2006. *Int J Equity Health*. 2011;10:12
11. Jayatunga W, Asaria M, Belloni A, George A, Bourne T, Sadique Z. Social gradients in health and social care costs: Analysis of linked electronic health records in Kent, UK. Public Health. 2019;169:188-94.
12. Asaria M, Doran T, Cookson R. The costs of inequality: whole-population modelling study of lifetime inpatient hospital costs in the English National Health Service by level of neighbourhood deprivation. J Epidemiol Community Health. 2016;70(10):990-6.
13. Kelly E,StoyeG, Vera-Hernandez M. Public hospital spending in England: evidence from National Health Service administrative records. FiscStud 2016;37:433e59.
14. Glenngård AH. The international Healthcare Systems Profiles: Sweden, https://www.commonwealthfund.org/international-health-policy-center/countries/sweden(2020), (accessed 2021-05-07).
15. OECD (2020). Health at a Glance: Europe 2020. State of health in the EU cycle. OECD.
16. SOU 2020:80 (2020). Äldreomsorgen under pandemin. Delbetänkande från Coronakommissionen [The eldercare during the pandemic. Interim report from the Corona Commission].
17. Brooke HL, Talbäck M, Hörnblad J, Johansson LA, Ludvigsson JF, Druid H, Feychting M, Ljung R. The Swedish cause of death register. Eur J Epidemiol. 2017 Sep;32(9):765-773. doi: 10.1007/s10654-017-0316-1. Epub 2017 Oct 5. PMID: 28983736; PMCID: PMC5662659.
18. Garcia-Ptacek S, Kåreholt I, Cermakova P, Rizzuto D, Religa D, Eriksdotter M. Causes of Death According to Death Certificates in Individuals with Dementia: A Cohort from the Swedish Dementia Registry. Journal of the American Geriatrics Society (JAGS). J Am Geriatr Soc 64:e137-e142, 2016. 2016;64(11):e137–e142
19. SBU. Hemvård – en kartläggning av översikter. [cited 5 Sep 2021] Available from: https://www.sbu.se/sv/publikationer/SBU-utvarderar/hemvard--en-kartlaggning-av-oversikter/
20. Ludvigsson JF, Appelros P, Askling J, Byberg L, Carrero JJ, Ekström AM, et al. Adaptation of the Charlson Comorbidity Index for Register-Based Research in Sweden. Clin Epidemiol. 2021;13:21-41.
21. Koenker, R. Quantile Regression, Cambridge University Press, 2005. Chp 2: (27-67).

For peer review only - http://bmjopen.bmj.com/site/about/guidelines.xhtml

22. Stoltzfus JC, Nishijima D, Melnikow J. Why quantile regression makes good sense for analyzinge conomic outcomes in medical research. AcadEmergMed 2012;19(7): 850-1
23. Mohammadpour S, Niknam N, Javan-Noughabi J, et al. The Factors Associated With Direct Medical Costs in Patients With Gastric Cancer: Quantile Regression Approach Compared With Gamma Regression. Value Health Reg Issues. 2020;21:127-132.
24. Lo TK, Parkinson L, Cunich M, Byles J. Factors associated with higher healthcare costs in individuals living with arthritis: evidence from the quantile regression approach. Expert Rev Pharmacoecon Outcomes Res. 2015;15(5):833-41.
25. Olsen, Margaret A et al. "Use of Quantile Regression to Determine the Impact on Total Health Care Costs of Surgical Site Infections Following Common Ambulatory Procedures." *Annals of surgery* vol. 265,2 (2017): 331-339.
26. Yu CW, Alavinia SM, Alter DA. Impact of socioeconomic status on end-of-life costs: a systematic review and meta-analysis. *BMC Palliat Care*. 2020;19(1):35.
27. Charlson M, Wells MT, Ullman R, King F, Shmukler C. The Charlson comorbidity index can be used prospectively to identify patients who will incur high future costs. PLoSOne. 2014;9(12):e112479.
28. Geue C, Briggs A, Lewsey J, Lorgelly P. Population ageing and healthcare expenditure projections: new evidence from a time to death approach. Eur J Health Econ. 2014;15(8):885-96.
29. Kelfve S, Wastesson J, Fors S, Johnell K, Morin L. Is the level of education associated with transitions between care settings in older adults near the end of life? A nationwide, retrospective cohort study. *Palliat Med*. 2018;32(2):366-375.
30. Gomes B, Calanzani N, Gysels M, Hall S, Higginson I. Heterogeneity and changes in preferences for dying at home: a systematic review. BMC palliative care. 2013;12(1):7–7
31. Hollander MJ, Chappell NL. A comparative analysis of costs to government for home care and long-term residential care services, standardized for client care needs. *Can J Aging*. 2007;26 Suppl 1:149-161.
32. Hallgren J, Johansson L, Lannering C, Ernsth Bravell M, Gillsjö C. Health- and social care in the last year of life among older adults in Sweden. BMC Palliat Care. 2020;19(1):90.
33. Swedish Agency for Health and Care Services Analysis. Perspective on health care among 65 years and older patients - International Comparison [Vården ur patienternas perspektiv - 65 år och äldre]. Stockholm: Vårdanalys; 2018.
34. Bone AE, Evans CJ, Etkind SN, Sleeman KE, Gomes B, Aldridge M, et al. Factors associated with older people's emergency department attendance towards the end of life: a systematic review. European journal of public health. 2019;29(1):67–74
35. Doheny M, Agerholm J, Orsini N, Schön P, Burström B. Socio-demographic differences in the frequent use of emergency department care by older persons: a population-based study in Stockholm County. *BMC Health Serv Res*. 2019;19(1):202. Published 2019 Mar 29. doi:10.1186/s12913-019-4029-x
36. Qureshi D, Isenberg S, Tanuseputro P, Moineddin R, Quinn K, Meaney C, et al. Describing the characteristics and healthcare use of high-cost acute care users at the end of life: a pan-Canadian population-based study. BMC Health Serv Res. 2020;20(1):997.
37. Schön P, Lagergren M, Kåreholt I. Rapid decrease in length of stay in institutional care for older people in Sweden between 2006 and 2012: results from a population-based study. Health Soc Care Community. 2016;24(5):631-8.
38. Lperovitch, A., Bertrand, M., Jougla, E., Vidal, J.-S., Ducimetiere, P., Helmer, C., Ritchie, K., Pavillon, G., and Tzourio, C. Do We Really Know the Cause of Death of the Very Old? Comparison between Official Mortality Statistics and Cohort Study Classification. European journal of epidemiology. 2009;24(11):669–75.

For peer review only - http://bmjopen.bmj.com/site/about/guidelines.xhtml

STROBE Statement—Checklist of items that should be included in reports of ***cohort studies***

	Item No	Recommendation	Page No
**Title and abstract**	1	(*a*) Indicate the study's design with a commonly used term in the title or the abstract<br>(*b*) Provide in the abstract an informative and balanced summary of what was done and what was found	2
**Introduction**			
Background/rationale	2	Explain the scientific background and rationale for the investigation being reported	3
Objectives	3	State specific objectives, including any prespecified hypotheses	4
**Methods**			
Study design	4	Present key elements of study design early in the paper	4
Setting	5	Describe the setting, locations, and relevant dates, including periods of recruitment, exposure, follow-up, and data collection	4
Participants	6	(*a*) Give the eligibility criteria, and the sources and methods of selection of participants. Describe methods of follow-up<br>(*b*) For matched studies, give matching criteria and number of exposed and unexpose	4
Variables	7	Clearly define all outcomes, exposures, predictors, potential confounders, and effect modifiers. Give diagnostic criteria, if applicable	4-5
Data sources/ measurement	8*	For each variable of interest, give sources of data and details of methods of assessment (measurement). Describe comparability of assessment methods if there is more than one group	5
Bias	9	Describe any efforts to address potential sources of bias	
Study size	10	Explain how the study size was arrived at	4
Quantitative variables	11	Explain how quantitative variables were handled in the analyses. If applicable, describe which groupings were chosen and why	5
Statistical methods	12	(*a*) Describe all statistical methods, including those used to control for confounding<br>(*b*) Describe any methods used to examine subgroups and interactions<br>(*c*) Explain how missing data were addressed<br>(*d*) If applicable, explain how loss to follow-up was addressed<br>(*e*) Describe any sensitivity analyses	6
**Results**			
Participants	13*	(a) Report numbers of individuals at each stage of study—eg numbers potentially eligible, examined for eligibility, confirmed eligible, included in the study, completing follow-up, and analysed<br>(b) Give reasons for non-participation at each stage<br>(c) Consider use of a flow diagram	4
Descriptive data	14*	(a) Give characteristics of study participants (eg demographic, clinical, social) and information on exposures and potential confounders<br>(b) Indicate number of participants with missing data for each variable of interest<br>(c) Summarise follow-up time (eg, average and total amount)	6
Outcome data	15*	Report numbers of outcome events or summary measures over time	6

For peer review only - http://bmjopen.bmj.com/site/about/guidelines.xhtml

Main results	16	(*a*) Give unadjusted estimates and, if applicable, confounder-adjusted estimates and their precision (eg, 95% confidence interval). Make clear which confounders were adjusted for and why they were included<br>(*b*) Report category boundaries when continuous variables were categorized<br>(*c*) If relevant, consider translating estimates of relative risk into absolute risk for a meaningful time period	6-11
Other analyses	17	Report other analyses done—eg analyses of subgroups and interactions, and sensitivity analyses	10-11
**Discussion**			
Key results	18	Summarise key results with reference to study objectives	11
Limitations	19	Discuss limitations of the study, taking into account sources of potential bias or imprecision. Discuss both direction and magnitude of any potential bias	14
Interpretation	20	Give a cautious overall interpretation of results considering objectives, limitations, multiplicity of analyses, results from similar studies, and other relevant evidence	12-14
Generalisability	21	Discuss the generalisability (external validity) of the study results	14
**Other information**			
Funding	22	Give the source of funding and the role of the funders for the present study and, if applicable, for the original study on which the present article is based	15

*Give information separately for exposed and unexposed groups.

Note: An Explanation and Elaboration article discusses each checklist item and gives methodological background and published examples of transparent reporting. The STROBE checklist is best used in conjunction with this article (freely available on the Web sites of PLoS Medicine at http://www.plosmedicine.org/, Annals of Internal Medicine at http://www.annals.org/, and Epidemiology at http://www.epidem.com/). Information on the STROBE Initiative is available at http://www.strobe-statement.org.

For peer review only - http://bmjopen.bmj.com/site/about/guidelines.xhtml

BMJ Open

Socio-economic differences in inpatient care expenditure in the last year of life among older people: A retrospective population-based study in Stockholm County

Journal:	*BMJ Open*
Manuscript ID	bmjopen-2022-060981.R1
Article Type:	Original research
Date Submitted by the Author:	04-May-2022
Complete List of Authors:	Doheny, Megan; Karolinska Institutet, Global Public Health<br>Schön, Pär; Aging Research Center<br>Orsini, Nicola; Karolinska Institutet, Global Public Health<br>Walander, Anders; Region Stockholm, Center for Epidemiology and Community Medicine<br>Burström, Bo; Karolinska Institutet, Global Public Health<br>Agerholm, J; Aging Research Center
**Primary Subject Heading**:	Health services research
Secondary Subject Heading:	Health policy
Keywords:	SOCIAL MEDICINE, HEALTH SERVICES ADMINISTRATION & MANAGEMENT, Health policy < HEALTH SERVICES ADMINISTRATION & MANAGEMENT, PUBLIC HEALTH

SCHOLARONE™ Manuscripts

I, the Submitting Author has the right to grant and does grant on behalf of all authors of the Work (as defined in the below author licence), an exclusive licence and/or a non-exclusive licence for contributions from authors who are: i) UK Crown employees; ii) where BMJ has agreed a CC-BY licence shall apply, and/or iii) in accordance with the terms applicable for US Federal Government officers or employees acting as part of their official duties; on a worldwide, perpetual, irrevocable, royalty-free basis to BMJ Publishing Group Ltd ("BMJ") its licensees and where the relevant Journal is co-owned by BMJ to the co-owners of the Journal, to publish the Work in this journal and any other BMJ products and to exploit all rights, as set out in our licence.

The Submitting Author accepts and understands that any supply made under these terms is made by BMJ to the Submitting Author unless you are acting as an employee on behalf of your employer or a postgraduate student of an affiliated institution which is paying any applicable article publishing charge ("APC") for Open Access articles. Where the Submitting Author wishes to make the Work available on an Open Access basis (and intends to pay the relevant APC), the terms of reuse of such Open Access shall be governed by a Creative Commons licence – details of these licences and which Creative Commons licence will apply to this Work are set out in our licence referred to above.

Other than as permitted in any relevant BMJ Author's Self Archiving Policies, I confirm this Work has not been accepted for publication elsewhere, is not being considered for publication elsewhere and does not duplicate material already published. I confirm all authors consent to publication of this Work and authorise the granting of this licence.

For peer review only

Socio-economic differences in inpatient care expenditure in the last year of life among older people: A retrospective population-based study in Stockholm County

Megan Doheny

Department of Global Public Health, Karolinska Institutet, Stockholm, SwedenAging Research Center, Karolinska Institutet - Stockholm University, Stockholm, Sweden

Pär Schön

Aging Research Center, Karolinska Institutet - Stockholm University, Stockholm, Sweden
Department of Social Work, Stockholm University, Stockholm, Sweden
par.schon@ki.se

Nicola Orsini

Department of Global Public Health, Karolinska Institutet, Stockholm, Sweden;
nicola.orsini@ki.se

Anders Walander

Center for epidemiology and community medicine, Region Stockholm, Sweden.
anders.walander@regionstockholm.se

Bo Burström

Department of Global Public Health, Karolinska Institutet, Stockholm, Sweden.
Center for epidemiology and community medicine, Region Stockholm, Sweden.
bo.burstrom@ki.se

Janne Agerholm

Aging Research Center, Karolinska Institutet - Stockholm University, Stockholm, Sweden
janne.agerholm@ki.se

Corresponding author: Megan Doheny
Orcid !D: 0000-0002-5640-1239
Address: Aging Research Center, Karolinska Institutet, Widerströmska plan 9, 171 71, Stockholm, Sweden.
E-mail: megan.doheny@ki.se
Telephone: +46 7 2548 6112

For peer review only

For peer review only

For peer review only - http://bmjopen.bmj.com/site/about/guidelines.xhtml

ABSTRACT

Objectives: To investigate the association between inpatient care expenditure (ICE) and income group and the effect of demographic factors, health status, healthcare, and social care utilization on ICE in the last year of life.

Design: Retrospective population-based study

Setting: Stockholm County.

Participants: Decedents ≥65 years in 2015 (N=13,538).

Outcome: ICE was calculated individually for the month of, and 12 months preceding death using healthcare register data from 2014 and 2015. ICE included the costs of admission and treatment in inpatient care adjusted for the price level in 2018.

Results: There were difference between income groups and ICE incurred at the 75th percentile, while a social gradient was found at the95th percentile where the highest income group incurred higher ICE (45,307SEK CI95% 12,055; 79,559) compared to the lowest income groups. Incurring higher ICE at the 95th percentile was driven by greater morbidity (20,333 SEK CI95% 12,673; 29,993) and emergency department care visits (77,995 SEK CI95% 64,442; 79,549), while lower ICE across the distribution was associated with older age and residing in institutional care.

Conclusion: Gaining insight into patterns of healthcare expenditure in the last year of life has important implications for policy, particularly as socio-economic differences were visible in ICE at a time of greater care need for all. Future policies should focus on engaging in advanced care planning and strengthening the coordination of care for older people.

Strengths and Limitations

- The quantile regression has flexible assumptions compatible with cost data and assesses the relationship between factors across the distribution of ICE.
- In this study, it was only feasible to measure ICE; however, which does not reflect total care costs that are accrued in the last year of life.
- Measuring need of care is difficult, particularly, during the last year of life as need changes.

Keywords

End-of-life, inpatient care expenditure, health and social care need, socio-economic differences, ageing in place.

Abstract: 299 words

Main text: 3,708 words

For peer review only

INTRODUCTION

An ageing population is often considered the main driver of increasing healthcare expenditure due to increasing prevalence of multi-morbidity (2+ chronic conditions) and more complex health problems [1]. However, age itself is not the main driver of healthcare expenditure but rather proximity to death, as a large proportion of all deaths occur among those 65 years and older [2-4], and a substantial proportion of an individual's lifetime healthcare expenditure occurs during the last year of life [5].

Patterns of healthcare utilisation in the last year of life often involve high rates of acute hospital-based services [6]. Congruently, a country comparison study of the composition of end-of-life (EOL) expenditure across nine different countries showed that expenditure on hospital-based inpatient care accounted for the largest proportion of total expenditure, followed by the utilisation of social care [7]. Additionally, a Swedish study observed that inpatient care specialties accounted for 80% of state expenditure on healthcare in the last year of life [8].

Often high spending on healthcare among older persons is not due to the use of more expensive life-saving treatments, but rather due to spending on treating persons with multiple chronic conditions [5]. Generally, healthcare utilisation and as such healthcare expenditure is largely influenced by the need of care, and health status which varies by socio-economic position (SEP). Frequently, older persons with lower SEP experience poorer health and have greater need of hospital-based care at the EOL which would lead to decedents with lower SEP incurring higher ICE [9]. However, previous studies have observed socio-economic differences in healthcare expenditure, where persons with higher SEP have higher expenditure compared to those with lower SEP [8, 10-13].

The Swedish healthcare and social care system is universal and primarily financed through taxes collected by the regions and municipalities. Additionally, the provision of care is based on the principle of "equal access for equal need" regardless of an individual's age, sex, or economic resources. However, the organization of care is decentralized where the 21 Regions are responsible for health and medical care and the 290 municipalities are responsible for social care for older people (home-help services and institutional care) [14]. Patient fees account for a small fraction of healthcare costs (3-5%), and moreover, there are cost ceilings for out-of-pocket patient fees, set at 1,105 SEK on healthcare visits during a 12-month period, when this threshold is exceeded, there is no further patient fees for the proceeding 12-months.

For peer review only

The out-of-pocket costs of an inpatient care stay is 100 SEK per day per adult [14]. So accordingly, a recent OECD report showed that Sweden has one of the highest average healthcare expenditures per capita, 4,676 euros. On the other hand, there is on average 2.1 hospital beds per 1,000 persons in Sweden which is among the lowest in OECD [15]. In municipal institutional care there is onsite health and medical care being provided by nurses, assistant nurses, and Primary Health Care (PHC) doctors, in addition to care personnel that provide daily personal care (bathing, eating, and hygiene), social activities and companionship in institutional care settings [16]. Home-help services can be offered around the clock and provides two main types of support: household tasks (e.g., cleaning and laundry, shopping, cooking or meals on wheels) and personal care. In parallel to the reduction in hospital-beds, there has been a similar decrease in the number of places in municipal institutional care, in Sweden. These changes have been brought about as cost containment strategies driven by the "Ageing in place" policy, which has resulted in an increasing number of older persons dependent on receiving care and support in their own homes [16].

Increasing the knowledge of the drivers of ICE and investigating whether there are socio-economic differences is important considering the ageing population with greater care needs and the goal of providing equitable care. This study aimed to investigate the association between inpatient care expenditure (ICE) and socio-economic position and the effect of other socio-demographic factors, health status, healthcare, and social care utilization on ICE in the last year of life.

METHODS

The study population was retrospectively identified, including those 65 years and older that died in the calendar year 2015 in Stockholm County (N=13,538). The last year of life was defined as the month of death plus the 12 months preceding the month of death. The Cause of Death register used to identify the study population using the year and month, to measure the age of death, place of death and the underlying cause of death [17]. The place of death is categorized into dying in a hospital, institutions/specialized geriatric clinics, private residence or other. The underlying cause of death categories were cancer-related, cardiovascular-related, neurodegenerative-related (including dementia, Alzheimer's, Parkinson's disease) and other causes [18].

Data sources

For peer review only

For peer review only - http://bmjopen.bmj.com/site/about/guidelines.xhtml

The Region Stockholm Healthcare Administrative Databases (VAL by Swedish acronym) inpatient care register was used to measure the outcome Inpatient Care Expenditure (ICE) which included the costs of admission (planned and unplanned) and treatment in inpatient care for acute somatic, geriatric, surgical and psychiatric departments. ICE was calculated for 2014 and 2015, adjusted for the price level in 2018 in Swedish Kronor (SEK), (10 SEK = 0.98 euros). The outcome was total ICE for each decedent measured as their total inpatient expenditures accrued from their month of death plus 12 months preceding death.

Socio-demographic characteristics of decedents were obtained from the Longitudinal Integration database for Health Insurance and Labour Market Studies (LISA). This register contains a collection of variables from different population registers linked individually via encrypted serial numbers. We measured sex, country of birth, living situation, and income in LISA. Sex was grouped as male and female. Country of birth was dichotomized as born in Sweden or born outside of Sweden. Living situation was measured for decedents living in the community and categorised as cohabiting or living alone. SEP was measured using income and assessed using the net annual equalized individual household income from 2012 and then ranked into income quintiles. Those missing a measure for income were excluded from the main analysis (n=56).

Healthcare utilisation during the last year life was obtained from VAL. PHC use was measured by the number of visits to GPs in outpatient care, categorised into 0-5 visits, 6-10 visits and >10 visits. Emergency department (ED) visits were defined as a registered emergency care visit to acute care hospitals in Stockholm County, and categorized into 0-1 visits, 2-3 visits and 4+ visits (frequent ED use). Both measures included in the regression analyses as continuous count variables.

Home-healthcare utilisation was measured in VAL as a period of being enrolled in receiving a period of basic-or advanced home- healthcare provided in a patient's home. There are two levels, basic and advanced which are provided based on medical need. Basic home-healthcare is provided to those that require simpler medical interventions up to nursing-level, such as assistance with taking medication, dressing of wounds, catheter replacement, or to those who have difficulties visiting GPs. Basic home-healthcare is free for all patients enrolled and those 85 years and older. Advanced home-healthcare is provided to seriously ill patients in need and involves medical procedures performed at home rather than in a hospital setting, often provided to cancer patients or those with complex EOL care needs and is not subject to patient fees [19].

For peer review only

To measure morbidity prior to death we calculated the Charlson Comorbidity Index (CCI) using registered diagnoses in VAL prior to the last year of life. The CCI assigns scores ranging from 1 to 6 to morbidities based on the severity of illness and risk of death [20], the CCI was calculated per decedent and was described in categories: 1-2 score, 3-4 score, and 5+ score, and was included as a continuous variable in regression analysis.

The utilisation of municipal social care was measured in the Swedish Social Services Register which collects data on use of social care on monthly basis. We identified those receiving home-help (personal care and/or domestic services) in their own homes as well as individuals registered as living in an institution for the entire 12-month period. Additionally, there was a group of individuals that were receiving home-help services in their own homes in the community, who moved into institutional care during their last year of life, hereafter, this group is referred to as the transition group. In the regression analysis the utilisation of home-help and institutional care was measured by number of months of use in the last year of life.

Patient and public involvement

The data used in this study was based on encrypted personal numbers so that the individuals in the study population are not identifiable.

Statistical Analysis

The quantile regression (QR) was selected to investigate how ICE varied between income groups (SEP) and to identify factors that affect the ICE incurred in the last year of life, because the dependent variable ICE has a positively skewed distribution with extreme outliers [21, 22]. The QR model estimates the change in a specified quantile (i.e., percentiles) of the conditional distribution of ICE due to a unit change in the independent variable. The QR was used to assess which independent variables are associated with quantiles of expenditure and has been recommended for the analysis of expenditure outcomes compared to alternative approaches in previous studies [23-25]. We estimated ICE at the 50th, 75th, and 95th percentiles, adjusting for the following explanatory variables (income, sex, age, country of birth, CCI score, ED use, home-healthcare and municipal social care use). These percentiles were selected as it was presumed that the rate of change in ICE per unit change in an explanatory variable ($\Delta ICE/\Delta x$) would be progressively greater in the higher percentiles. There was n=2,912 decedents who incurred zero ICE in the last year of life. A logistic regression model was used assess whether there were socio-economic differences among those that incurred zero ICE in the last year of life.

For peer review only - http://bmjopen.bmj.com/site/about/guidelines.xhtml

RESULTS

There were N=13,538 decedents included in the study population described in **table 1**. Most decedents (51.7%) were 85+ years, (54.2%) female and (25.7%) in income group 3. Most decedents in the community were living alone (63.4%), and 18.5% were born outside of Sweden. There were 38.8% of decedents who had a cardiovascular-related death and 25.3% with a cancer-related underlying cause of death. During the last year of life, decedents had on average 4.5 PHC visits and 2.7 ED visits. Further, 28.6% of decedents frequently attended ED care (4+ visits) in the last year of life. Most decedents utilized municipal social care, 32.5% received home-help services, 28.3% were in institutional care, and 10.5% had transition from receiving home-help into an institution during the last year of life.

Those in the lowest income group incurred higher costs at the 50th percentile (139,401 SEK) compared to other income groups, while at the 75th and 95th percentile those in the highest income group incurred higher ICE (287,722 SEK at 75th) and (669,124 SEK at 95th) compared to the lowest income group (281,968 SEK at 75th) and (633,349 SEK at 95th) respectively. There was an inverse relationship between age and ICE, where those 85+ years incurred lower ICE compared to those 65-74 years. Females incurred lower ICE compared to males at the 50th, 75th and 95th percentiles, a similar pattern could be observed among those living alone and those born outside of Sweden.

For peer review only

For peer review only - http://bmjopen.bmj.com/site/about/guidelines.xhtml

Table 1: Description of decedents 65+ years, and the distribution of of Inpatient Care Expenditure (ICE) in the last year of life

	N	(%)	Percentiles of Inpatient Care Expenditure (SEKs) (median) 50th percentile	75th percentile	(highest) 95th percentile
	13,538	100	129,886	279,152	644,596
**Income group**					
Group 1 (Lowest)	2,647	19.6	139,401	281,968	633,349
Group 2	3,160	23.3	131,404	284,388	656,418
Group 3	3,475	25.7	120,833	260,599	617,288
Group 4	2,517	18.6	132,311	291,504	660,780
Group 5 (highest)	1,683	12.4	132,203	287,722	669,124
**Age in years**	83.8(9.0)				
65 - 74 years	2,672	19.7	161,587	358,466	903,567
75 - 84 years	3,875	28.6	158,874	321,478	721,371
85+ years	6,991	51.7	109,658	231,026	506,885
**Sex**					
Male	6,204	45.8	147,442	306,065	702,300
Female	7,334	54.2	116,217	257,460	591,401
**Country of birth**					
Sweden	10,963	81.5	131,695	281,814	647,807
Outside of Sweden	2,489	18.5	128,031	275,844	624,877
**Living situation**					
Cohabiting	3,544	36.6	168,215	322,015	700,553
Alone	6,132	63.4	115,251	259,849	613,316
**Healthcare utilisation**					
**Average no. PHC visits**	4.5 (6.9)				
0–5 PHC visits	9,753	72.0	104,608	239,889	585,186
6–10 PHC visits	2,011	14.9	191,834	345,298	735,614
>10 PHC visits	1,774	13.1	216,465	387,845	804,472
**Average no. ED visits**	2.7 (3.0)				
0–1 ED visits	5,460	40.3	25,433	88,392	299,568
2–3 ED visits	4,208	31.1	156,467	248,957	522,653
>4 ED visits	3,870	28.6	317,664	481,791	870,365
**Home-health care**					
Basic care	3,602	26.6	225,700	382,229	759,464
Advanced care	1,651	12.2	202,885	355,234	718,165
**Social care utilisation**					
Independent	3,863	28.5	125,057	269,586	696,304
Home-help	4,393	32.5	203,872	359,895	721,371
Institutional care	3,862	28.5	41,823	130,698	392,649
Transition	1,420	10.5	202,336	346,695	710,872
**Months with home-help**	8,2(4.9)				
**Months in institutional care**	9.0(3.8)				
**Health status and Underlying cause of death**					
**CCI score**	3.1(2.6)				
0 - 2 score	6,878	50.8	60,216	163,026	451,067
3 - 4 score	2,873	21.2	188,170	341,629	697,367
5 + score	3,787	28.0	236,137	406,819	805,941
**Cause of Death**					
Cancer-related	3,425	25.3	195,908	343,042	681,738

For peer review only - http://bmjopen.bmj.com/site/about/guidelines.xhtml

Cardiovasuclar-related	5,221	38.6	119,816	274,621	654,265
Neurodegenerative related	1,995	14.7	45,393	143,758	396,939
Other	2,897	21.4	132,820	288,010	686,942
**Place of Death**					
Hospital	5,853	43.2	203,204	362,355	780,051
Institution/specialised geriatric clinic	4,807	25.5	69,464	192,532	484,093
Private residence	1,918	14.2	68,809	209,693	552,571
Other	960	7.1	85,155	240,580	600,078

*Variables living situation and home-healthcare utilization are measures applicable to those living in the community for in the last 12-months of life (n=9,676).

Decedents that had more visits to PHC and other outpatient care services incurred higher ICE. Those receiving basic home-healthcare incurred higher ICE compared to those receiving advanced home-healthcare. Decedents receiving home-help services or that transitioned to institutional care incurred higher ICE compared to those independent. Persons with cancer-related deaths incurred higher ICE at the 50th, 75th and 95th percentiles, while those with neurodegenerative-related cause of death incurred lower ICE. Most decedents (43.2%) died in hospital, 25.5% died in institutional care and 14.2% died in a private residence. Those that died in hospital incurred higher ICE compared to the other places of death.

Quantile Regression

Table 2 contains the estimates from the QR analysis. We observed socio-economic differences in ICE incurred at the 75th and 95th percentile by income group. Those in the second, third- and fourth -income group incurred higher ICE at the 75th percentile compared to the lowest income group. There was a social gradient in the ICE incurred at the 95th percentile as those in higher income groups incurred higher ICE. Those in the highest income group incurred (45,307SEK CI95% 12,055; 79,559) higher ICE compared to those in the lowest income group at the 95th percentile.

.

For peer review only - http://bmjopen.bmj.com/site/about/guidelines.xhtml
For peer review only

Table 2: Quantile regression (QR) estimates conditional on the 50th (median), 75th and 95th (high-cost patients). of the distribution of Inpatient Care Expenditure in the last year of life among all decedents 65+ years in Stockholm County.

	50th percentile		75th percentile		95th percentile	
	Coefficient (SEK)	CI 95%	Coefficient (SEK)	CI 95%	Coefficient (SEK)	CI 95%
Income group						
Group 1 (lowest)	ref		ref		ref	
Group 2	1,925	-474 – 4,323	10,032	4,714 – 15,350	41,720	14,824–68,615
Group 3	-520	-3,201 – 2,161	9,354	3,273 – 15,436	43,252	19,263–67,240
Group 4	304	-2,468 – 3,076	11,704	4,495 – 18,913	42,762	11,875–73,650
Group 5 (highest)	-912	-3,805 – 1,981	8,360	-817 – 17,537	45,307	12,055–78,559
Age in years	-304	-469 – 139	-1,672	-2,154 – -1,190	-9,761	-12,070– -7,452
Sex						
Male	ref		ref		ref	
Female	2,635	-145 –5,414	5,126	-1,384 – 11,636	11,614	-11,220–34,449
Country of birth						
Sweden	ref		ref		Ref	
Outside of Sweden	-2,736	-4,367 – -1,104	-11,318	-15,785 – -6,851	-21,054	-41,726–-382
CCI score	12,358	11,180 – 13,537	16,720	14,999 – 18,441	20,333	12,673–27,993
ED visits	42,586	41,002 – 44,170	57,362	54,463 – 60,260	71,995	64,442–79,549
Home-healthacre						
None	Ref		ref		ref	
Basic care	31,017	23,791 – 38,243	36,040	21,050 – 51,029	15,299	-23,046 – 55,643
Advanced care	11,412	2,777 – 20,048	34,179	20,382 – 47,976	-32,009	-82,581 – 18,563
Months of home-help	475	-54 – 1,005	456	-677 –1,589	-4,216	-7,386 – -1,045
Months of institutional care	-945	-1,117 – -774	-2,074	-2,741– -1,408	-11,593	-15,136 – -8,050
Intercept	24,620	11,015 – 38,224	163,558	122,071 – 205,545	1,061,896	855,819 – 1,267,972

*QR model adjusted for income group (ref=lowest income group 1), age in years, sex (ref=male), country of birth (ref=Sweden), CCI score, ED visits, Home-healthcare (ref=no care in the community, months in institutional care and months receiving home-help.

For peer review only - http://bmjopen.bmj.com/site/about/guidelines.xhtml

Increasing age in years was associated with lower ICE at the 50th, 75th and 95th percentile. There were no significant differences between sexes. Decedents born outside of Sweden incurred lower ICE at the 50th (-2,736SEK CI95%-4,367; -1,104) and at the 75th percentile (-11,318SEK CI95% -15,785; -6,851) compared to decedents born in Sweden. Greater morbidity and more visits to ED care were positively associated with incurring higher ICE. ED visits were a significant driver for higher ICE, an increase in the number ED visits was associated with (71,995 SEK CI 95% 64,442; 79,549) higher ICE at the 95th percentile. Those receiving basic home-healthcare incurred higher ICE at the 50th and 75th percentiles, there was similar pattern with advanced home healthcare but slightly lower at the 75th percentile (34,179 SEK CI95% 21,050; 51,029). Months in institutional care were associated with incurring lower ICE across the distribution, while additional months with home-help were associated with lower ICE (-4,216 SEK CI95% -7,1386; -1,045) at the 95th percentile

Zero Expenditure

There were 2,912 decedents that incurred zero ICE, 56.6% of decedents with zero costs resided in institutional care and 29.9% lived independently. Those with zero cost living in the community had an average age of death of 78.4 years, were mostly male, 26.5% born outside of Sweden, utilised more PHC than ED care in the last year of life. Most of those with zero ICE (53.2%) had cardiovascular-related death and 53.6% died in private residence. Table 3 shows models estimating odds of incurring zero ICE among decedents that were living in the community, there were no socio-economic differences observed in the odds of zero ICE, while those born outside of Sweden and had a cardiovascular-related underlying cause of death had higher odds of zero ICE. Older age and the utilisation of healthcare and social care were associated lower odds of having zero ICE.

Table 3: Logistic regression estimates the odds of incurring zero inpatient care costs in the last year of life.

	All Zeroes	Community Dwelling Decedents						
	N=2,912	N=1,254	Model 1			Model 2		
			OR	CI95%		OR	CI95%	
Income group								
(lowest) Group 1	17.2%	20.9%	ref			ref		
Group 2	24.1%	21.5%	1.02	0.77	1.36	0.96	0.74	1.25
Group 3	27.9%	24.6%	0.93	0.70	1.24	0.90	0.69	1.18
Group 4	18.5%	19.4%	0.95	0.70	1.28	0.95	0.72	1.26
(highest) Group 5	12.2%	13.6%	1.01	0.72	1.40	0.99	0.73	1.35
**Age in years**	84.9 (9.9)	78.6 (9.1)						
65 - 74 years	19.4%	39.6%	ref			ref		
75 - 84 years	22.7%	30.3%	0.86	0.68	1.07	0.87	0.71	1.08

For peer review only

85 + years	57.8%	30.1%	0.71	0.56	0.90	0.80	0.64	1.00
**Sex**								
Male	39.2%	54.3%	ref			ref		
female	60.8%	45.7%	0.90	0.75	1.09	0.96	0.80	1.15
**Living situation**								
Cohabiting			ref			ref		
Living alone	69.1%	69.1%	1.13	0.91	1.41	1.23	1.00	1.50
**Country of Birth**								
Sweden			ref			ref		
Outside of Sweden	19.5%	26.5%	1.42	1.13	1.77	1.39	1.13	1.70
**CCI score**	1.1(1.6)	1.0 (1.9)	0.59	0.56	0.62			
**Healthcare utilisation**								
Average no. ED visits	0.3 (0.7)	0.4 (0.8)	0.17	0.15	0.19	0.14	0.13	0.16
Average no. PHC visits	1.9 (4.8)	4.3 (6.5)	0.99	0.97	1.00			
**Home-healthcare**								
none			ref			ref		
Basic	5.2%	12.0%	0.55	0.42	0.71	0.44	0.35	0.57
Advanced	4.3%	10.0%	1.04	0.77	1.42	0.70	0.51	0.95
**Underlying Cause of Death**								
Other	21.6%	25.2%				ref		
Cancer-related	8.4%	15.3%				0.29	0.22	0.39
Cardiovascular -related	41.3%	53.2%				1.30	1.05	1.60
Neurodegenerative-related	28.7%	6.3%				1.21	0.84	1.72
**Place of Death**								
Hospital	7.9%	16.3%						
Institution/ geriatric specialised clinic	57.1%	10.8%						
Private residence	23.5%	53.6%						
Unknown	11.5%	19.3%						
**Social care utilisation**								
Independent	29.9%	68.8%						
Home-help	10.5%	24.2%						
Institutional care	56.6%	.						
Transition	3.0%	7.0%						
**Months with home-help**			0.98	0.96	1.00	0.97	0.95	0.99

*Community-Dwelling decendents refers all individuals living in their own in the community with or without home-help services *Column one the description of those with zero ICE in the last year of life in proportions (%) and mean and standard deviation. *OR=odds ratio, CI95% =95% confidence interval ref=indicating the reference group, *Logistic regression models stratified into the group of persons with zero costs living in the community n=1,254 for the most of their last year of life.***Model 1** is adjusted for (income group, age groups, sex, country of birth, living situation, phc visits, ED visits, home-healthcare, CCI score, months with home-help).***Model 2** is adjusted for (income group, age groups, sex, country of birth, living situation, PHC visits, ED visits, home-healthcare, underlying cause of death, months with home-help).

For peer review only

For peer review only - http://bmjopen.bmj.com/site/about/guidelines.xhtml

DISCUSSION

This study investigated the socio-economic differences in inpatient care expenditure (ICE) in the last year of life and assessed the effect of other demographic factors, health status, healthcare and social care utilisation had on ICE. We observed that decedents in the higher income group incurred higher ICE at the 75th percentile, and there was a social gradient in ICE at the 95th percentile with higher ICE in the higher income groups. Older age was associated with incurring lower ICE across the distribution. Greater morbidity and visits to ED care were positively associated with incurring higher ICE across the distribution. Months in institutional care was associated with incurring lower ICE overall, while months with home-help were associated with lower ICE at the 95th percentile.

Based on findings from earlier studies, we expected that older people with lower SEP due to greater need would utilise more healthcare and incur greater ICE [8-13, 21]. Previous studies from the UK observed that persons in the most deprived quintile incurred higher inpatient care costs than the least deprived [12], similar findings were observed in other studies on those 65 years and older [11, 13]. Our findings are akin with a previous Swedish study that found the persons in the highest income group had higher healthcare expenditure in the last year of life compared to the lowest [8]. In contrast to this study, Hanratty et al included adults of all ages and measured total healthcare expenditure (inpatient and outpatient costs) and yet, we observed similar socio-economic differences despite these deviations in study population and outcome [8].

Poorer health and functioning in the last year of life should be the main determinant of hospital use and subsequent expenditure on care. As a systematic review and meta-analysis found that socio-economic differences in EOL healthcare expenditure can vary based on adjustment for need of care [26]. We took this into consideration and included the CCI score as an indicator of "need" measured prior to the last year of life. The CCI score has been demonstrated to be effective at predicting persons who will incur high healthcare costs [27]. The socio-economic differences were observed at the top 5% of the distribution and remained after adjusting for need, but whether these differences are indicating inequities in access or treatment cannot be determined in this study.

The socio-economic differences observed in this study may have many explanations. It could be due to more affluent and better educated individuals, or their families being better equipped to navigate the healthcare system and advocate for more extensive or expensive

For peer review only - http://bmjopen.bmj.com/site/about/guidelines.xhtml

EOL care. However, this pro-rich bias of hospital care has had mixed findings, a Scottish study that found there were no differences in the costs incurred by SEP once hospitalized, though differences were observed in when persons from more deprived areas reached hospital care [28]. Further, a Swedish study found that decedents 65+ years with tertiary education were more likely to die in hospital compared to those primary education [29]. This raises the questions about the appropriateness of EOL care in terms of the socioeconomic differences observed in ICE but also that most decedents died in hospital which is not the preferred place of death, as a systematic review reported that people prefer to die in their own homes, even as illness progresses [30]. Although, most community-dwelling decedents who incurred zero ICE in their last year of life died in private residence, it is difficult to discern whether this was their preference and if they received appropriate care in the last year of life.

Age was associated with incurring lower ICE in the last year of life, this finding is consistent with previous studies [3,4,6-13] and in line with the "Red Herring" theory, which stipulates that as age increases healthcare expenditure decreases and social care expenditure increases [2, 3]. Decedents residing in institutional care incurred lower ICE and a large proportion incurred zero ICE. This result might be due to the around-the-clock presence of health professionals and caregivers in institutional care settings, which may facilitate better EOL care. Previous studies observed that independent community-dwelling decedents were more frequently hospitalised and had higher healthcare costs compared to those in institutional care after standardizing for similar care needs [31]. Similarly, a Swedish study found that older people living in their own homes were hospitalized more frequently in the last 10 weeks of life [32].

Older people are now increasingly "ageing in place", and their care needs shall be met at home. There is a greater need for advanced care planning near the EOL, as most decedents in were receiving municipal social care and even those receiving home-healthcare incurred high ICE. A Swedish report comparing healthcare systems in ten different countries, highlighted that Sweden had deficits in care planning at the EOL [33]. ED visits were a driver of higher ICE, possibly due to subsequent unplanned hospitalisations and treatment in inpatient care [32]. However, if care received in the home is sufficiently meeting needs, as found in a systematic review, receiving appropriate palliative home-based care should lower ED use among dying patients [34]. Nevertheless, a previous Swedish study found that receiving home-healthcare was associated with frequent ED use [35].

For peer review only

Additionally, care transitions that occur in the last year of life are stressful for patients and family members as well as being costly and difficult to organise in the care system. A Canadian study found that high-cost acute care users had multiple care transitions during the last year of life and had longer hospital stays because of lack of places in institutional care or due to lack of availability of homecare to discharge patients [36]. The provision of social care is essential in the last year life and can reduce inpatient care costs. The de-institutionalisation trend might leave some patients in precarious living situations during a difficult time, as the overall length of stay in institutions is decreasing with a large proportion of people moving into institution and dying shortly afterward, in Sweden [37].

Strengths and Limitations

A strength of this study is the use of high-quality register data, that allowed us to retrospectively follow our study population and measure their ICE and their utilisation of other health and social care services in last year of life. The QR model allowed us to assess the relationship between SEP and other factors across the distribution of ICE from lowest to the highest, as the QR model has flexible assumptions [21, 22]. The Swedish Cause of Death Register provides complete coverage of the population, and we have measures for all decedents [18]. However, as older persons often experience multi-morbidity deciphering the exact cause of death can be difficult especially among the very old [38]. Therefore, we also included the CCI score to indicate need [20], though we are limited in our ability to fully measure an individual's need and the extent that these needs change during the last year of life [5, 27]. Another limitation of this study is that we only focused on expenditure on inpatient care, which does not provide a complete overview of total costs accrued in the EOL, as social care accounts for a substantial proportion of EOL care and there are other costs such as for outpatient visits and pharmaceutical drugs [4, 7]. Future research should use a longitudinal approach and explore the socio-economic differences in patterns of health and social care services in the last year of life and the years prior to understand how socio-economic differences can emerge.

CONCLUSIONS

Most deaths occur in older age, and now most older people with complex health problems are receiving care in their own homes. Gaining insight into patterns of healthcare spending in the last year of life has important implications for policy, as socio-economic differences were visible in ICE at a time of greater care need for all, indicating inequity in end-of-life care.

For peer review only - http://bmjopen.bmj.com/site/about/guidelines.xhtml

Future policies should focus on engaging in advanced care planning and strengthening the coordination of care in older people homes.

For peer review only

For peer review only - http://bmjopen.bmj.com/site/about/guidelines.xhtml

CONTRIBUTION STATEMENT

MD, PS, AW, BB and JA conceived the idea for, participated in the design and the coordination of this study. MD performed the statistical analysis and drafted the manuscript. MD, JA, NO, PS and BB were all involved in the interpretation of results and the revisions of manuscript drafts. All authors read and approved the submitted manuscript.

FUNDING

The authors disclosed receipt of the following financial support for the research, authorship and/or publication of this article: The research leading to these results was carried out as part of the Social Inequalities in Ageing (SIA) project, funded by NordForsk project no. 74637, and funding was also provided by the Swedish Research Council for Health, Working Life and Welfare (Forte), grant numbers 2017-02155 and 2017-04431.

CONFLICT OF INTEREST

None

ETHICAL APPROVAL

Ethical permissions were obtained from the Regional Ethics Review Board of Stockholm (Dnr 2016/299–31).

PARTICIPANTS CONSENT

Individual consent to participate in this study is waived as this was a population-based study using administrative data.

DATA STATEMENT

The data used will not be shared, as the materials are based on individual data which cannot be shared in accordance with Swedish law and GDPR.

REFERENCES

1. SOU 2016:2. Effektiv vård. Slutbetänkande av En nationell samordnare för effektivare resursutnyttjande inom hälso- och sjukvården. Stockholm 2016 [Effective care. Final report by A national coordinator for more efficient use of resources in health care]
2. Zweifel, P., S. Felder, and M. Meiers (1999), 'Aging of population and health care expenditure: a red herring?', Health Economics, 8(6): 485–496.

For peer review only

3. Polder JJ, Barendregt JJ, van Oers H. Health care costs in the last year of life--the Dutch experience. Soc Sci Med. 2006;63(7):1720-31.
4. Jayatunga W, Lewer D, Shand J, Sheringham J, Morris S, George J. Health and social care costs at the end of life: a matched analysis of linked patient records in East London. Age Ageing. 2019;49(1):82-7.
5. Davis MA, Nallamothu BK, Banerjee M, Bynum JP. Identification Of Four Unique Spending Patterns Among Older Adults In The Last Year Of Life Challenges Standard Assumptions. Health Aff (Millwood). 2016;35(7):1316-23.
6. Ní Chróinín D, Goldsbury DE, Beveridge A, Davidson PM, Girgis A, Ingham N, et al. Health-services utilisation amongst older persons during the last year of life: a population-based study. BMC Geriatr. 2018;18(1):317.
7. French EB, McCauley J, Aragon M, Bakx P, Chalkley M, Chen SH, et al. End-Of-Life Medical Spending In Last Twelve Months Of Life Is Lower Than Previously Reported. HealthAff(Millwood). 2017;36(7):1211-7.
8. Hanratty B, Burström B, Walander A, Whitehead M. Inequality in the face of death? Public expenditure on health care for different socioeconomic groups in the last year of life. J HealthServRes Policy. 2007;12(2):90-4.
9. Davies JM, Maddocks M, Chua KC, Demakakos P, Sleeman KE, Murtagh FEM. Socioeconomic position and use of hospital-based care towards the end of life: a mediation analysis using the English Longitudinal Study of Ageing. Lancet Public Health. 2021;6(3):e155-e63.
10. Cunningham CM, Hanley GE, Morgan SG. Income inequities in end-of-life health care spending in British Columbia, Canada: A cross-sectional analysis, 2004-2006. *Int J Equity Health*. 2011;10:12
11. Jayatunga W, Asaria M, Belloni A, George A, Bourne T, Sadique Z. Social gradients in health and social care costs: Analysis of linked electronic health records in Kent, UK. Public Health. 2019;169:188-94.
12. Asaria M, Doran T, Cookson R. The costs of inequality: whole-population modelling study of lifetime inpatient hospital costs in the English National Health Service by level of neighbourhood deprivation. J Epidemiol Community Health. 2016;70(10):990-6.
13. Kelly E,StoyeG, Vera-Hernandez M. Public hospital spending in England: evidence from National Health Service administrative records. FiscStud 2016;37:433e59.
14. Glenngård AH. The international Healthcare Systems Profiles: Sweden, https://www.commonwealthfund.org/international-health-policy-center/countries/sweden(2020), (accessed 2021-05-07).
15. OECD (2020). Health at a Glance: Europe 2020. State of health in the EU cycle. OECD.
16. SOU 2020:80 (2020). Äldreomsorgen under pandemin. Delbetänkande från Coronakommissionen [The eldercare during the pandemic. Interim report from the Corona Commission].
17. Brooke HL, Talbäck M, Hörnblad J, Johansson LA, Ludvigsson JF, Druid H, Feychting M, Ljung R. The Swedish cause of death register. Eur J Epidemiol. 2017 Sep;32(9):765-773. doi: 10.1007/s10654-017-0316-1. Epub 2017 Oct 5. PMID: 28983736; PMCID: PMC5662659.
18. Garcia-Ptacek S, Kåreholt I, Cermakova P, Rizzuto D, Religa D, Eriksdotter M. Causes of Death According to Death Certificates in Individuals with Dementia: A Cohort from the Swedish Dementia Registry. Journal of the American Geriatrics Society (JAGS). J Am Geriatr Soc 64:e137-e142, 2016. 2016;64(11):e137–e142
19. SBU. Hemvård – en kartläggning av översikter. [cited 5 Sep 2021] Available from: https://www.sbu.se/sv/publikationer/SBU-utvarderar/hemvard--en-kartlaggning-av-oversikter/
20. Ludvigsson JF, Appelros P, Askling J, Byberg L, Carrero JJ, Ekström AM, et al. Adaptation of the Charlson Comorbidity Index for Register-Based Research in Sweden. Clin Epidemiol. 2021;13:21-41.
21. Koenker, R. Quantile Regression, Cambridge University Press, 2005. Chp 2: (27-67).
22. Stoltzfus JC, Nishijima D, Melnikow J. Why quantile regression makes good sense for analyzinge conomic outcomes in medical research. AcadEmergMed 2012;19(7): 850-1

For peer review only - http://bmjopen.bmj.com/site/about/guidelines.xhtml

23. Mohammadpour S, Niknam N, Javan-Noughabi J, et al. The Factors Associated With Direct Medical Costs in Patients With Gastric Cancer: Quantile Regression Approach Compared With Gamma Regression. Value Health Reg Issues. 2020;21:127-132.
24. Lo TK, Parkinson L, Cunich M, Byles J. Factors associated with higher healthcare costs in individuals living with arthritis: evidence from the quantile regression approach. Expert Rev Pharmacoecon Outcomes Res. 2015;15(5):833-41.
25. Olsen, Margaret A et al. "Use of Quantile Regression to Determine the Impact on Total Health Care Costs of Surgical Site Infections Following Common Ambulatory Procedures." *Annals of surgery* vol. 265,2 (2017): 331-339.
26. Yu CW, Alavinia SM, Alter DA. Impact of socioeconomic status on end-of-life costs: a systematic review and meta-analysis. *BMC Palliat Care*. 2020;19(1):35.
27. Charlson M, Wells MT, Ullman R, King F, Shmukler C. The Charlson comorbidity index can be used prospectively to identify patients who will incur high future costs. PLoSOne. 2014;9(12):e112479.
28. Geue C, Briggs A, Lewsey J, Lorgelly P. Population ageing and healthcare expenditure projections: new evidence from a time to death approach. Eur J Health Econ. 2014;15(8):885-96.
29. Kelfve S, Wastesson J, Fors S, Johnell K, Morin L. Is the level of education associated with transitions between care settings in older adults near the end of life? A nationwide, retrospective cohort study. *Palliat Med*. 2018;32(2):366-375.
30. Gomes B, Calanzani N, Gysels M, Hall S, Higginson I. Heterogeneity and changes in preferences for dying at home: a systematic review. BMC palliative care. 2013;12(1):7–7
31. Hollander MJ, Chappell NL. A comparative analysis of costs to government for home care and long-term residential care services, standardized for client care needs. *Can J Aging*. 2007;26 Suppl 1:149-161.
32. Hallgren J, Johansson L, Lannering C, Ernsth Bravell M, Gillsjö C. Health- and social care in the last year of life among older adults in Sweden. BMC Palliat Care. 2020;19(1):90.
33. Swedish Agency for Health and Care Services Analysis. Perspective on health care among 65 years and older patients - International Comparison [Vården ur patienternas perspektiv - 65 år och äldre]. Stockholm: Vårdanalys; 2018.
34. Bone AE, Evans CJ, Etkind SN, Sleeman KE, Gomes B, Aldridge M, et al. Factors associated with older people's emergency department attendance towards the end of life: a systematic review. European journal of public health. 2019;29(1):67–74
35. Doheny M, Agerholm J, Orsini N, Schön P, Burström B. Socio-demographic differences in the frequent use of emergency department care by older persons: a population-based study in Stockholm County. *BMC Health Serv Res*. 2019;19(1):202. Published 2019 Mar 29. doi:10.1186/s12913-019-4029-x
36. Qureshi D, Isenberg S, Tanuseputro P, Moineddin R, Quinn K, Meaney C, et al. Describing the characteristics and healthcare use of high-cost acute care users at the end of life: a pan-Canadian population-based study. BMC Health Serv Res. 2020;20(1):997.
37. Schön P, Lagergren M, Kåreholt I. Rapid decrease in length of stay in institutional care for older people in Sweden between 2006 and 2012: results from a population-based study. Health Soc Care Community. 2016;24(5):631-8.
38. Lperovitch, A., Bertrand, M., Jougla, E., Vidal, J.-S., Ducimetiere, P., Helmer, C., Ritchie, K., Pavillon, G., and Tzourio, C. Do We Really Know the Cause of Death of the Very Old? Comparison between Official Mortality Statistics and Cohort Study Classification. European journal of epidemiology. 2009;24(11):669–75.

For peer review only - http://bmjopen.bmj.com/site/about/guidelines.xhtml

STROBE Statement—Checklist of items that should be included in reports of ***cohort studies***

	Item No	Recommendation	Page No
**Title and abstract**	1	(*a*) Indicate the study's design with a commonly used term in the title or the abstract<br>(*b*) Provide in the abstract an informative and balanced summary of what was done and what was found	2
**Introduction**			
Background/rationale	2	Explain the scientific background and rationale for the investigation being reported	3
Objectives	3	State specific objectives, including any prespecified hypotheses	4
**Methods**			
Study design	4	Present key elements of study design early in the paper	4
Setting	5	Describe the setting, locations, and relevant dates, including periods of recruitment, exposure, follow-up, and data collection	4
Participants	6	(*a*) Give the eligibility criteria, and the sources and methods of selection of participants. Describe methods of follow-up<br>(*b*) For matched studies, give matching criteria and number of exposed and unexpose	4
Variables	7	Clearly define all outcomes, exposures, predictors, potential confounders, and effect modifiers. Give diagnostic criteria, if applicable	4-5
Data sources/ measurement	8*	For each variable of interest, give sources of data and details of methods of assessment (measurement). Describe comparability of assessment methods if there is more than one group	5
Bias	9	Describe any efforts to address potential sources of bias	
Study size	10	Explain how the study size was arrived at	4
Quantitative variables	11	Explain how quantitative variables were handled in the analyses. If applicable, describe which groupings were chosen and why	5
Statistical methods	12	(*a*) Describe all statistical methods, including those used to control for confounding<br>(*b*) Describe any methods used to examine subgroups and interactions<br>(*c*) Explain how missing data were addressed<br>(*d*) If applicable, explain how loss to follow-up was addressed<br>(*e*) Describe any sensitivity analyses	6
**Results**			
Participants	13*	(a) Report numbers of individuals at each stage of study—eg numbers potentially eligible, examined for eligibility, confirmed eligible, included in the study, completing follow-up, and analysed<br>(b) Give reasons for non-participation at each stage<br>(c) Consider use of a flow diagram	4
Descriptive data	14*	(a) Give characteristics of study participants (eg demographic, clinical, social) and information on exposures and potential confounders<br>(b) Indicate number of participants with missing data for each variable of interest<br>(c) Summarise follow-up time (eg, average and total amount)	6
Outcome data	15*	Report numbers of outcome events or summary measures over time	6

For peer review only

For peer review only - http://bmjopen.bmj.com/site/about/guidelines.xhtml

Main results	16	(*a*) Give unadjusted estimates and, if applicable, confounder-adjusted estimates and their precision (eg, 95% confidence interval). Make clear which confounders were adjusted for and why they were included<br>(*b*) Report category boundaries when continuous variables were categorized<br>(*c*) If relevant, consider translating estimates of relative risk into absolute risk for a meaningful time period	6-11
Other analyses	17	Report other analyses done—eg analyses of subgroups and interactions, and sensitivity analyses	10-11
**Discussion**			
Key results	18	Summarise key results with reference to study objectives	11
Limitations	19	Discuss limitations of the study, taking into account sources of potential bias or imprecision. Discuss both direction and magnitude of any potential bias	14
Interpretation	20	Give a cautious overall interpretation of results considering objectives, limitations, multiplicity of analyses, results from similar studies, and other relevant evidence	12-14
Generalisability	21	Discuss the generalisability (external validity) of the study results	14
**Other information**			
Funding	22	Give the source of funding and the role of the funders for the present study and, if applicable, for the original study on which the present article is based	15

*Give information separately for exposed and unexposed groups.

Note: An Explanation and Elaboration article discusses each checklist item and gives methodological background and published examples of transparent reporting. The STROBE checklist is best used in conjunction with this article (freely available on the Web sites of PLoS Medicine at http://www.plosmedicine.org/, Annals of Internal Medicine at http://www.annals.org/, and Epidemiology at http://www.epidem.com/). Information on the STROBE Initiative is available at http://www.strobe-statement.org.

For peer review only
